# Association of Chronic *Opisthorchis* Infestation and Microbiota Alteration on Tumorigenesis in Cholangiocarcinoma

**DOI:** 10.14309/ctg.0000000000000292

**Published:** 2020-12-22

**Authors:** Thanika Ketpueak, Parameth Thiennimitr, Nattayaporn Apaijai, Siriporn C. Chattipakorn, Nipon Chattipakorn

**Affiliations:** 1 Division of Oncology, Department of Internal Medicine, Faculty of Medicine, Chiang Mai University, Chiang Mai, Thailand;; 2 Department of Microbiology, Faculty of Medicine, Chiang Mai University, Chiang Mai, Thailand;; 3 Cardiac Electrophysiology Research and Training Center, Faculty of Medicine, Chiang Mai University, Chiang Mai, Thailand;; 4 Center of Excellence in Cardiac Electrophysiology Research, Chiang Mai University, Chiang Mai, Thailand;; 5 Cardiac Electrophysiology Unit, Department of Physiology, Faculty of Medicine, Chiang Mai University, Chiang Mai, Thailand.

## Abstract

Cholangiocarcinoma (CCA) is a common hepatobiliary cancer in East and Southeast Asia. The data of microbiota contribution in CCA are still unclear. Current available reports have demonstrated that an *Opisthorchis viverrini* (OV) infection leads to dysbiosis in the bile duct. An increase in the commensal bacteria *Helicobacter* spp. in OV-infected CCA patients is associated with bile duct inflammation, severity of bile duct fibrosis, and cholangiocyte proliferation. In addition, secondary bile acids, major microbial metabolites, can mediate cholangiocyte inflammation and proliferation in the liver. A range of samples from CCA patients (stool, bile, and tumor) showed different degrees of dysbiosis. The evidence from these samples suggests that OV infection is associated with alterations in microbiota and could potentially have a role in CCA. In this comprehensive review, reports from *in vitro*, *in vivo*, and clinical studies that demonstrate possible links between OV infection, microbiota, and CCA pathogenesis are summarized and discussed. Understanding these associations may pave ways for novel potential adjunct intervention in gut microbiota in CCA patients.

## INTRODUCTION

Cholangiocarcinoma (CCA) is one of the highest incidence and mortality cancers in East and Southeast Asia (1–3). According to the RARECARE project in 2013, high incident countries including Thailand, China, and South Korea have approximately 7.1–85/100,000 population cases/yr (4). CCA cases worldwide have shown a tendency to increase during the past decade (5). Currently, CCA patients still have poor prognosis because of the lack of effective treatment. CCA is classified into intrahepatic, perihilar, and distal CCA according to anatomical site, cell origination, and potential contributory factors (6). There are many known risk factors of CCA including parasitic infestations, *Opisthorchis viverrini* (OV), and *Clonorchis sinensis* (6–8). The prevalence of each liver fluke species including OV, *Opisthorchis felineus*, and *Clonorchis sinensis* is different among geographic region. Around 10 million of OV-infected people are found in countries among Makong river neighborhood which are Thailand, Laos, Cambodia, Myanmar, and Southern Vietnam (9), while *Opisthorchis felineus* is predominately found in North of Asia including China, South Korea, Northern Vietnam, and Eastern Russia and *Clonorchis sinensis* in Western Siberia and Russia (9). Adult liver flukes can pass through human host biliary tract and cause mechanical irritation, release parasitic toxic secretions, and activate the host immune response, ending up with bile duct inflammation, fibrosis, and CCA development (10,11). After ingestion of contaminated food such as uncooked fish, the liver fluke metacercariae encyst in gastrointestinal tract. Adult liver flukes can pass ampulla of Vater through human host biliary tract. There are 3 main mechanism to CCA pathogenesis after this infection (12). The first is mechanical irritation from feeding and migrating. The second mechanism is parasitic excretion/secretion products which contained hundreds of proteins such as granulin and thioredoxin. Granulin and thioredoxin can promote fibroblast and cholangiocyte proliferation and suppress apoptosis (11–13). The third mechanism is immunopathology that drives by chronic inflammation. Liver fluke and its eggs can trigger inflammation cascade, and interleukin (IL)-6 is the main proinflammatory cytokines elevation responsible in this setting (10,14). So, all of that end up with bile duct inflammation, fibrosis, and CCA development.

The microbiota are described as a nonpathogenic microbial community living inside the human body (15,16). The interactions between these microorganisms and their human host are important in several host physiologies (17–19). The gut microbiota composition in each individual could be shaped by both internal and external factors, including genetics, diet, geography, stress, and drugs (20). An alteration in gut microbiota or gut dysbiosis has been reported as an underlying condition of many noncommunicable diseases including obesity and metabolic syndrome (21). In hepatobiliary disease, specifically non-alcoholic steatohepatitis and liver cirrhosis, Lipopolysaccharide (LPS)-containing bacteria are also abundant (22–24). The impaired gut barrier integrity from gut inflammation allows for LPS-containing bacteria, especially Gram-negative bacteria in the phylum *Proteobacteria*, to translocate from the gut lumen to gut tissue. Subsequently, a systemic immune response to this potent immunogenic bacterial LPS is triggered. Systemic inflammation can occur before the development of insulin resistance and liver steatosis (25).

There is a growing body of evidence to demonstrate that there is a correlation between the microbiota and both antitumor and protumor effects on various cancer cells. For example, *Propionibacterium* could induce colorectal cell apoptosis through short-chain fatty acid production (26). However, an increase in the numbers of LPS-containing bacteria including *Escherichia coli* and *Helicobacter* spp. has been shown to be associated with hepatocellular carcinoma development (27,28). Bacterial LPS can be recognized by innate immune receptors and toll-like receptor–4, located on the apical surface of several types of epithelia (29). Toll-like receptor–4 activation results in chronic inflammation, tumor proliferation, and reduced mononuclear antitumor activity in hepatocellular carcinoma, gastric cancer, and colorectal cancer (29–32). Potential roles of microbiota in cancer therapy have been demonstrated. For example, microbial LPS have been shown to activate dendritic cells and tumor-specific T cells to accentuate an improved radiation effect on melanoma treatment in T-lymphocyte–depleted mice (33). Also, microbiota with probiotic properties, e.g., *Bifidobacterium* spp., exhibited antitumoral activity by improving the efficacy of anti–programmed cell death 1 antibodies in mice inoculated with melanoma and bladder cancer cells (34).

In this review, currently available basic and clinical reports on the association between microbiota from various sources and CCA are summarized and discussed. Despite the limited number of pertinent resources, the information provided in this review could allow us to understand the contribution of microbiota profiles to CCA carcinogenesis with the hope that the overview could provide useful information for future CCA prevention, prognostic prediction, and improved treatment.

### Microbiota alterations during an OV infection: evidence from *in vivo* reports

Although the relationship between an OV infection and CCA is well-established (7), the link between the OV infection, CCA, and microbiota is still unclear. There is growing evidence to demonstrate the influence of OV infection on alterations in microbiota. In hamsters fed with OV metacercariae, the infective larval stage, an increased α-diversity of gut microbiota and increased bacteria in phylum *Firmicutes* (*Ruminococcaceae*, *Lacnospiracea*, and *Lactobacillus*) were found in the stool samples (35). Comparing the α-diversity between different specimens, a microbial diversity within a niche was greater in the stool than the bile of the hamsters (35). The bacterial community also differed between tissue sites. In the bile of OV-fed hamsters and OV worms, *Propionibacterineae*, *Lactobacillus*, and many other species in the phylum *Proteobacteria* such as *Burkholderia*, *Enterobacteriaceae*, and *Archea* were abundant (35). However, this profile was not detected in the stools (35). In liver tissues, only *Bifidobacterium*, *Escherichia*, and *Helicobacter* were found in the OV-infected hamsters (36). One of the most abundant species in OV-infected hamsters, *Aggregatibacter*, was also identified in the gut of OV worms. Since microbiota in OV worms are similar to that in the stools of OV-infected hamsters, it is possible that after an OV infection, the microbiota could be directly transferred from OV worm into the bile duct of the host and reside in the host's biliary system or liver. Furthermore, *Helicobacter* spp. could be detected in both the stools and liver tissue of the OV-infected mice especially *Helicobacter pylori* and *H. bilis* (36,37). To eliminate *Helicobacter* and *Opisthorchis*, antibiotics and antiparasitic agents were studied to identify any augmented efficacy. The number of *Helicobacter* species in OV-infected mice was diminished after treatment with antibiotics and further decreased after both antibiotics and antiparasitic agents were given (37). These findings indicated the association between OV and *Helicobacter*. A summary of these reports is shown in Table 1.

**Table 1.**
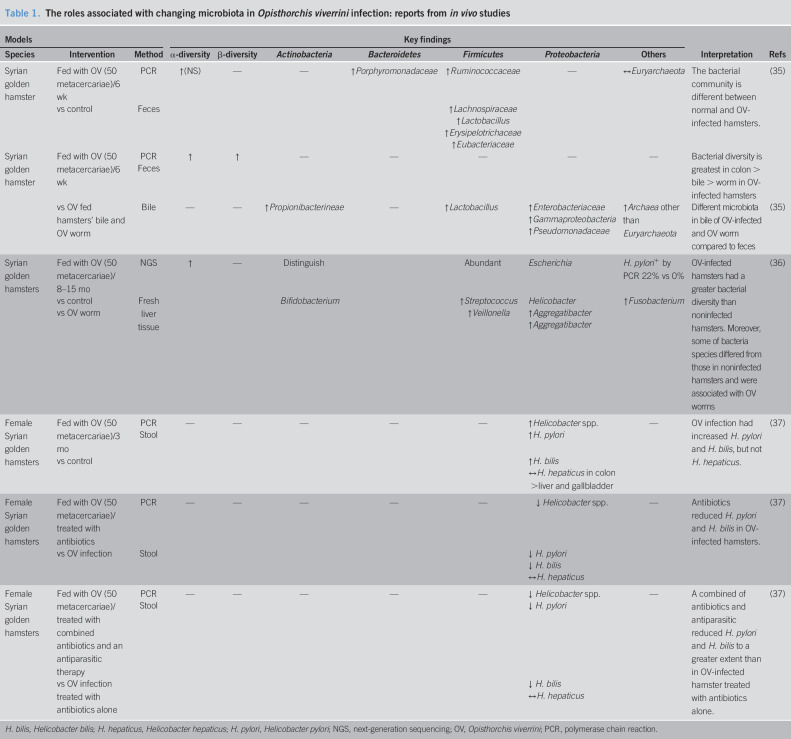
The roles associated with changing microbiota in *Opisthorchis viverrini* infection: reports from *in vivo* studies

### The inflammatory alteration in liver tissue after OV and *Helicobacter* infection: evidence from *in vivo* reports

Hamsters have been used as a study model for OV infection. In a hamster model of OV infection, it has been shown that there was an increase in bile duct fibrosis and the severity of cholangitis, without changes in inflammatory markers or animal survival rate (38). In hamsters coinfected with OV and *H. pylori*, increased levels of IL-1, α-smooth muscle antibodies (α-SMA), and transformation growth factor beta (TGF-β) were demonstrated, leading to further accumulation of inflammatory cells, and increased bile duct and liver fibrosis, finally resulting in decreased animal survival rate, compared with control (38). Comparing coinfection with the *H. pylori* monoinfection, increased IL-1, α-SMA, and TGF-β were observed, resulting in increased bile duct and liver fibrosis but no change in inflammatory responses. However, comparing coinfection with the OV-monoinfected hamsters, only profibrotic TGF-β and α-SMA were increased in the liver without additional liver fibrosis detected. These findings suggested that both organisms could synergistically contribute to chronic inflammation, thus aggravating the severity of fibrosis of bile duct and liver. A summary of these reports is shown in Table 2.

**Table 2.**
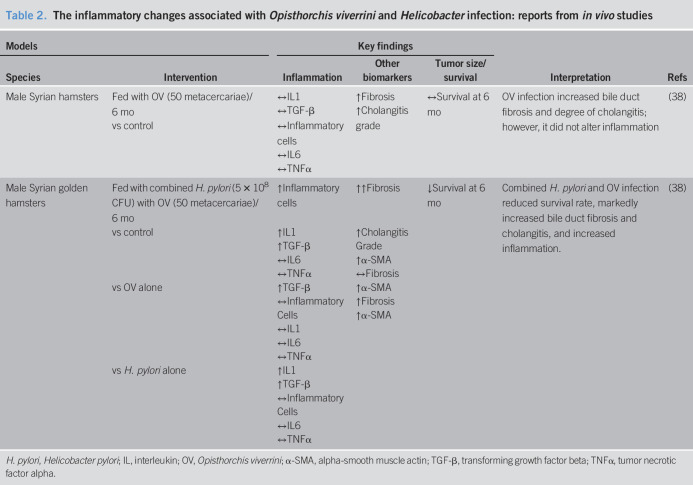
The inflammatory changes associated with *Opisthorchis viverrini* and *Helicobacter* infection: reports from *in vivo* studies

### Chronic OV infection results in *Helicobacter* overgrowth in humans: evidence from clinical reports

**Figure 1.**
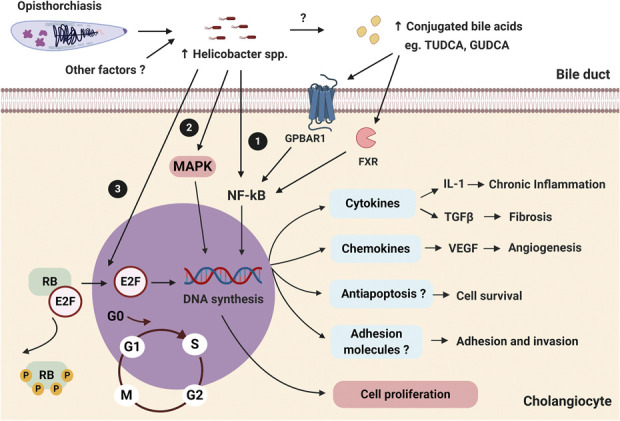
Microbiota alteration contributes to CCA carcinogenesis. Increased levels of commensal *Helicobacter* spp. during a parasitic OV infection result in chronic inflammation and abnormal cell proliferation of cholangiocytes. (1) Increased levels of conjugated bile acids (TUDCA and GUDCA) within the bile duct lumen from the increases in *Helicobacter* spp. lead to cholangiocyte inflammation through the NF-kB pathway. Proinflammatory cytokine IL-1, profibrotic cytokine TGF-β, and angiogenetic vascular endothelial growth factor are upregulated in cholangiocytes. (2) *Helicobacter* spp. can activate the mitogen-activated protein kinase pathway resulting in cholangiocyte proliferation. (3) *Helicobacte*r spp. can dysregulate the cell cycle of cholangiocytes by phosphorylation of the RB, a tumor-suppressing protein, and then release transcription factor E2F resulting in abnormal cholangiocyte proliferation. E2F, E2 factor; GUDCA, glycoursodeoxycholic acid; NF-kB, nuclear factor-kappa B; OV, *Opisthorchis viverrini*; RB, retinoblastoma; TUDCA, tauroursodeoxycholic acid.

There is growing evidence to show potential roles of *Helicobactor* spp. in people with chronic OV infection. In OV-infected people, it has been shown that the number of *Helicobacter* spp. found in the stools was higher in comparison with that of non–OV-infected people (39). OV infection status was identified by OV egg detection in stool samples (39). With regard to the diversity of *Helicobacter* spp., the presence of *cagA* and *cagE* genes in *H. pylori* was associated with high virulence (40). When *H. pylori* attaches to epithelial cells, CagA and CagE proteins are translocated to the plasma membrane, and cause phosphorylation of the SRC family kinase, thus resulting in many signal transductions in the mammalian host (41). Importantly, CagA can activate SRC homology 2-domain-containing protein tyrosine phosphatase, a human oncoprotein, to promote carcinogenesis especially in gastric cancer (41,42). It has been shown that the *cagA + cagE + H. pylori* is higher in the OV-infected population compared with that of a non–OV-infected population (39). In addition, the presence of *cagA + H. pylori* was associated with biliary periductal fibrosis with a significant relative risk ratio 3.38 (39). However, currently, the mechanism of this pathogen overgrowth in OV-infected humans is still under explored. Nevertheless, it has been shown in CCA cells that coculture with *cagA + Helicobacter* spp. led to increased antiapoptotic bcl-2 and activated mitogen-activated protein kinase and nuclear factor-kappa B (NF-kB) pathways, resulting in further biliary cancer cell proliferation (43). Moreover, *cagA + Helicobacter* spp. stimulated IL-8 production, leading to biliary cell inflammation. The proposed carcinogenesis mechanisms caused by OV infection and *Helicobacter* spp. are shown in Figure 1.

### Microbiota changes in the gut, bile, and cancer tissue and their roles in CCA patients

Microbial diversity and composition vary in accordance with their niches (15). The specimen collection from different sites is one of the findings highlighted in this review. There is evidence to demonstrate that microbiota can be found not only in the gut, but also bile and cancer tissue. In addition, dysbiosis differs between the various specimen sites in CCA patients.

#### Gut dysbiosis in CCA patients.

The gastrointestinal tract is the largest site with the highest density and variety of microbiota (15,44). Stool samples can be used as a noninvasive specimen that, at least in some parts, indicates the individual gut microbiota. The results of clinical studies into the changes in gut microbiota in CCA patients are summarized in Table 3. Comparisons of (i) intrahepatic CCA; (ii) hepatocellular carcinoma; (iii) liver cirrhosis; and (iv) healthy individuals found that CCA patients had the highest species richness (α-diversity) (45). A number of *Lactobacillus*, *Actinomyces*, *Peptostreptococcaceae*, *Alloscardovia*, and *Bifidobacteriaceae* were markedly increased in the stools of CCA patients (45). In addition, vascular invasion in CCA pathology, which is considered a poor prognostic factor, was associated with high levels of *Ruminococcaceae* species in the stools and IL-4 in the plasma (45). Information regarding gut microbiota alteration in CAA is not clearly understood at this time. This review collected and summarized all of involving reports available to date. Available information suggested that there was an association between microbiota alteration and cholangiocarcinoma including liver fluke infection. However, there is still a gap of knowledge whether changes in gut microbiota are the cause of the tumor. Future studies with rigorous study design are needed to answer whether the microbiota changes are also the risk factor of CCA.

**Table 3.**
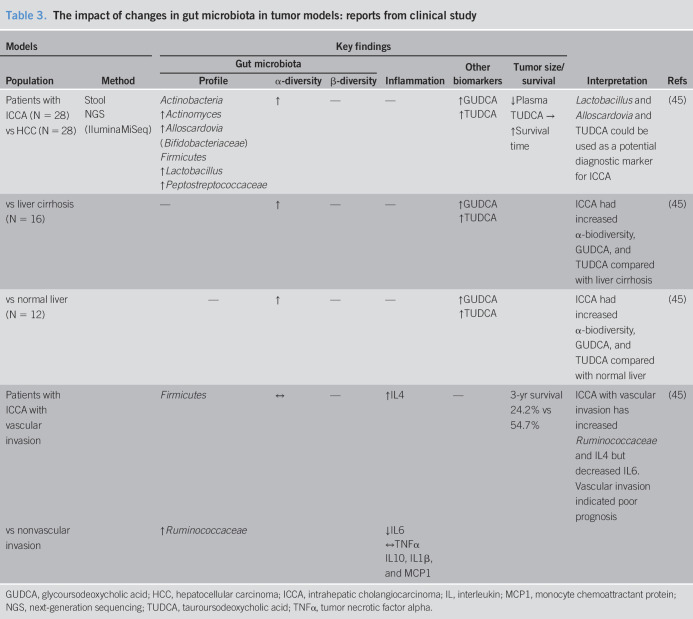
The impact of changes in gut microbiota in tumor models: reports from clinical study

**Figure 2.**
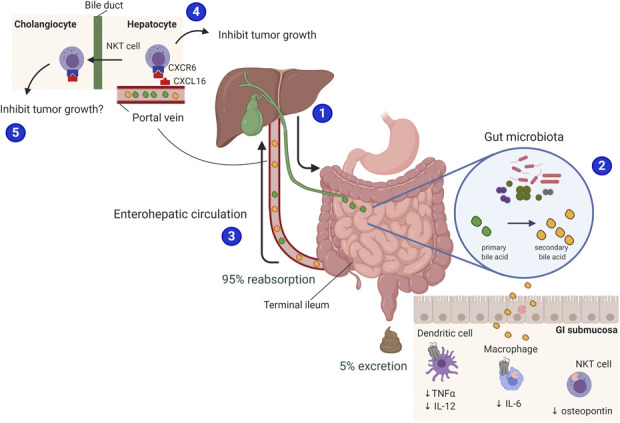
Association of biliary pathway and host immunity. (1) Primary bile acids (cholic acid and chenodeoxycholic acid) are mainly synthesized and conjugated with glycine and taurine respectively in the liver and are then released into the gastrointestinal tract. (2) In the intestinal lumen, gut microbiota metabolize these primary bile acids to secondary bile acids (deoxycholic and lithocholic acid, respectively). Secondary bile acids can regulate immune response in gut by decreasing proinflammatory cytokines. (3) Subsequently, enterohepatic circulation reabsorbs 95% of bile acids in the terminal ileum, and they are deconjugated in the liver through the portal vein. Nonreabsorbed secondary bile acids (5%) in the terminal ileum will be then excreted in the feces. (4) In the liver, deconjugated secondary bile acids become primary bile acids that could inhibit hepatic tumor growth by the induction of CXCL16-activated NKT cells. (5) However, it is uncertain whether these primary bile acids are able to inhibit tumor growth in the bile duct. CXCL16, C-X-C Motif Chemokine Ligand 16; FXR, farnesoid X receptors; G + PBAR1, G protein-coupled bile acid receptor 1; NKT cells, natural killer T cells.

Bile acids and their derivatives, mostly resulting from the metabolism of the gut microbiota, have been proposed as important mediators connecting the role of gut microbiota to the pathogenesis of CCA (46,47). In the bile acid metabolism pathway, primary bile acids (cholic acid and chenodeoxycholic acid) are synthesized in the liver and conjugated into a water-soluble form (48). Then, these bile acids enter the gastrointestinal tract and are metabolized by the microbiota in the intestine to become secondary bile acids (deoxycholic and lithocholic acid) (48). These secondary bile acids are then reabsorbed in the terminal section of the ileum and being transported back to the liver and deconjugated within liver tissues (48). In the gut lamina propria, secondary bile acids exert an anti-inflammatory effect by inhibiting proinflammatory cytokine production by macrophages, dendritic cells, and dampened the function of natural killer T cells (49). In addition, primary bile acids were shown to activate natural killer T cells through C-X-C Motif Chemokine Ligand 16 and inhibited tumor growth in the liver (50). The action of bile acids in tumor growth inhibition is shown in Figure 2.

Conjugated bile acids, e.g., glycocholic acid, glycodeoxcholic acid, or glycochenodeoxycholic acid, could promote tumorigenesis, whereas unconjugated bile acids including cholic acid, deoxycholic acid, or chenodeoxycholic acid could inhibit CCA cell proliferation (47). In CCA patients, the conjugated forms of the secondary bile acids glycoursodeoxycholic acid and tauroursodeoxycholic acid were elevated in the plasma/stool ratio (45). Moreover, analyzing bile acid from bile specimen among malignant liver cancer, nonmalignant liver disease, and nonliver disease, liver cancer could be distinguished from nonmalignant liver disease. Although glycine-conjugated bile acid was significantly increased in hepatocellular carcinoma, both glycine-conjugated bile acid and taurine-conjugated bile acid were also elevated in CCA without statistical significance (51). These findings suggested that an alteration of the gut microbiota in CCA could lead to the changes in the levels of the secondary bile acids. Both conjugated bile acids affected cholangiocyte proliferation by the activation of NF-kB signaling which is the transcription factor responsible for instigating several pathways (47,52). All these findings suggested that an increase in either secondary or conjugated bile acids could be a factor that promotes CCA carcinogenesis. However, the role of bile acids needs more supported studies to explain the association between bile acids and dysbiosis in CCA patients. The combination of bile acids and a microbiota profile could be used as a new biomarker in CCA. Detection of plasma tauroursodeoxycholic acid together with the presence of *Lactobacillus* and *Alloscardovia* in the stools could be used as a biomarker for the differentiation in intrahepatic cholangiocarcinoma from other liver pathologies (45). Since the standard diagnosis of CCA remains liver biopsy to obtain tumor tissue pathology, this noninvasive method could be a potential diagnostic biomarker for intrahepatic cholangiocarcinoma. These reports are summarized in Table 3.

#### Bile microbiota changes in CCA patients.

Since the biliary system connects with gut microbiota through enterohepatic circulation, evaluation of bile microbiota could determine the link between gut and bile microbiota in CCA. There was a report in healthy subjects without previous hepatobiliary disease (53). Bile acid was obtained from liver transplant donor during the operation. Bacteria in phylum *Firmicutes*, *Bacteroidetes*, *Actinobacteria*, and *Proteobacteria* were found in bile of healthy subjects. Therefore, this evidence suggested of bile microbiota existence in healthy individuals. Under the pathological condition, the changes in bile acid microbiota and/or the microbiota composition were observed. However, it is not known whether these changes occurred before or after the development of the disease since there is no solid evidence to indicate whether microbiota changes are secondary to tumor development. To answer this question will require stronger evidence with different timepoint investigation to explain whether this is just the association or the causation. Conventionally, microbiota assay was performed by culture-dependent method from the bile collected by an endoscopic retrograde cholangiopancreatography (ERCP) procedure. In hepatobiliary diseases, increased levels of *Klebsiella pneumoniae* in bile were shown to have a positive correlation with CCA (54).

Using a specific polymerase chain reaction to detect *Helicobacter* spp. from bile specimens, it has been shown that the positive detection of *Helicobacter* spp. including *non–H. pylori* species was higher in bile of CCA patients than that in the benign biliary tract disease patients (55–59). Moreover, *cagA + H. pylori*, the highly virulent and oncogenic strain of *H. pylori*, was increased in the bile of CCA patients, compared with that of cholelithitic or healthy individuals (56). The presence of *Helicobacter* spp. was also found to be associated with increased proliferation of the cell nuclear antigen and a biliary inflammatory histopathological score in CCA patients (59). All these findings suggest that the presence of *Helicobacter* spp. in the bile of CCA patients could be an important risk factor for tumor development through ongoing chronic inflammation. Moreover, assessment of bile microbiota from ERCP by quantitative polymerase chain reaction showed an increase in species richness in the distal CCA patients compared with that of cholelithiatic patients. Such abundant species including *Gemmatimonadetes*, *Nitrospirae*, *Chloroflexi*, *Latescibacteria*, and *Planctomycetes* (60). A summary of these reports is shown in Table 4.

**Table 4.**
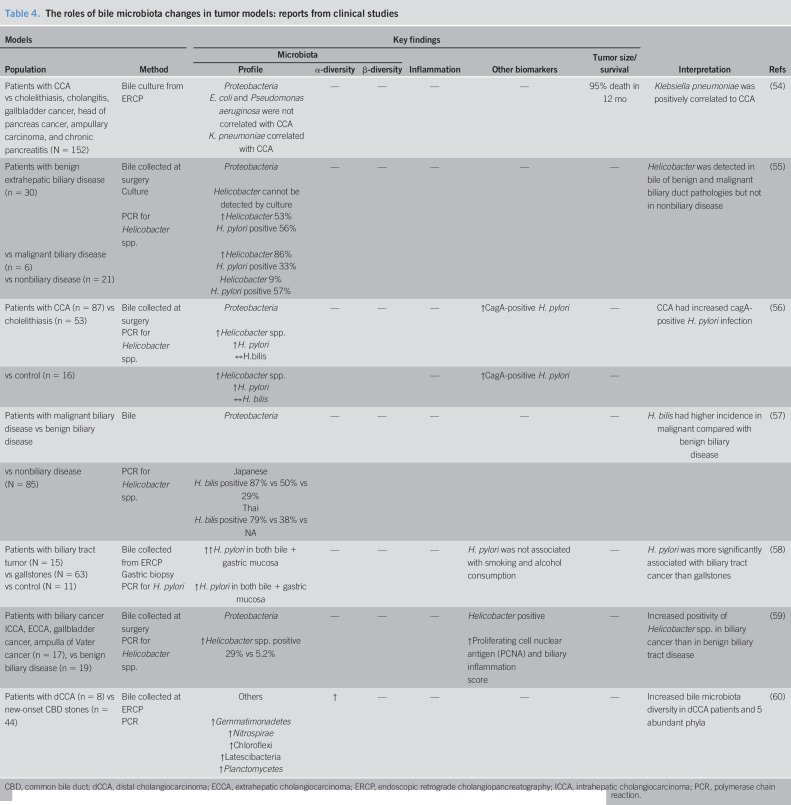
The roles of bile microbiota changes in tumor models: reports from clinical studies

CBD, common bile duct; dCCA, distal cholangiocarcinoma; ECCA, extrahepatic cholangiocarcinoma; ERCP, endoscopic retrograde cholangiopancreatography; ICCA, intrahepatic cholangiocarcinoma; PCR, polymerase chain reaction.

#### Cancer tissue microbiota changes in CCA patients.

Analysis of the microbiota has been reported after specific polymerase chain reaction in the tumor tissue obtained from invasive procedures such as surgery or ERCP cytobrush in CCA patients. Tissue-specific microbiota can be more accurate as regards identification of chronic colonization than bile-specific microbiota which may be transient contaminants during the collecting procedure (59). An elevation of 3 major species of *Helicobacter* spp. including *H. pylori*, *Helicobacter bilis*, and *Helicobacter hepaticus* has been reported in CCA patients (59,61–63). The pathological evaluation also demonstrated an increase in inflammation and Ki-67, an indicator of cell mitosis, in CCA patients with *Helicobacter* infection (56,59). These findings are consistent with an *in vitro* report demonstrating that biliary tract cancer cell line incubated with *H. bilis* had increased activity of NF-kB, E2F, a cyclic adenosine monophosphate response element and vascular endothelial growth factor (64). These findings indicated that *Helicobacter* infestation was associated with a proliferative pathway activation and angiogenesis upregulation, contributing to CCA (64). The potential relationship between carcinogenesis and changes in microbiota is also shown in Figure 1.

The culture-independent methods, such as next-generation sequencing, have extensively transformed the microbiota research in the past decades. By using next-generation sequencing, several “nonculturable” or “difficult to be cultured” bacterial species such as *Methylophilaceae*, *Sinobacteriaceae*, *Actinomyces*, *Dialister*, *Novosphingobium*, *Prevotella*, *Fusobacterium*, and high virulent *H. pylori* have been shown to be elevated in the tumor tissue of CCA patients (65). Although those families of microbiota came from different phyla, it emphasized the importance of *H. pylori* as the species which most jeopardizes CCA.

**Figure 3.**
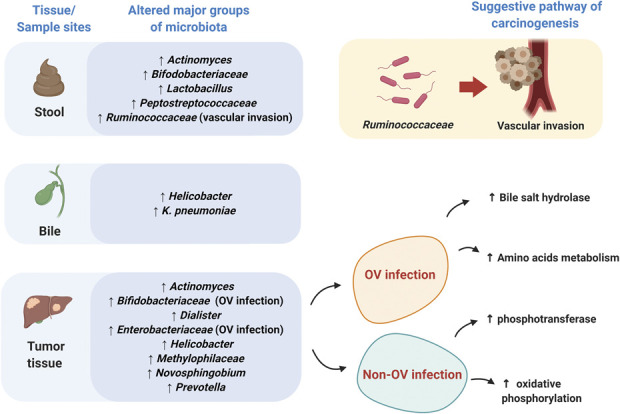
Different microbiota populations found in different tissue sites of CCA patients. Samples from different tissue sites illustrate the variations in microbiota profile as shown.

In CCA patients, an OV infection was also directly correlated with increased *Bifidobacteriaceae* and *Enterobacteriaceae* (66). It has been proposed that an OV infection altered the host metabolism. Increased amino acid metabolism and bile salt hydrolase was demonstrated in OV-infected tumor tissue (66). In the non–OV-infected group in this study, the phosphotransferase system and oxidative phosphorylation in tumor tissue were increased representing increased cell energy production (66). These reports indicated that the key pathogenesis is completely diverse among those populations. However, microbiota alteration was associated with high oncogenicity together with an OV infection. Interestingly, a coinfection with OV leads to increased *Bifidobacterium* spp. in the tumor tissue in CCA patients. *Bifidobacterium* spp. produce a bile salt hydrolase enzyme which changes primary to secondary bile acids, mediates the gelatinous process of bile, and also degrades bile acids into amino acids (67,68). Amino acid metabolites from bile acids due to *Bifidobacterium* can be detected in tumor tissue of OV-infected CCA patient (66). These findings suggested that *Bifidobacterium* might play a major role in the alteration in host metabolism during an OV infection and as such contributes to CCA development. Moreover, in opisthorchiasis, a proinflammatory cytokine IL-6 was increased, leading to biliary periductal fibrosis (14,69). However, this phenomenon was not found in some individuals infected with OV (14,69). This could be due to other factors such as dysbiosis or levels of bile acids which could be contributing factors in the inflammatory status in CCA patients coinfected with OV. Analysis of pathological tissue and adjacent normal liver tissue was studied to identify any variation in microbiota. In an individual patient, α-diversity was similar in tumor tissue and adjacent normal tissue (66). There was no difference in abundance of different species in OV-infected CCA tissue. Nevertheless, *Stenotrophomonas* and *Xanthomonadaceae* were increased in non–OV-infected CCA tissue compared with the adjacent normal tissue. A summary of these reports is shown in Table 5. A summary of species abundance in different sites of specimen collection is shown in Figure 3.

**Table 5.**
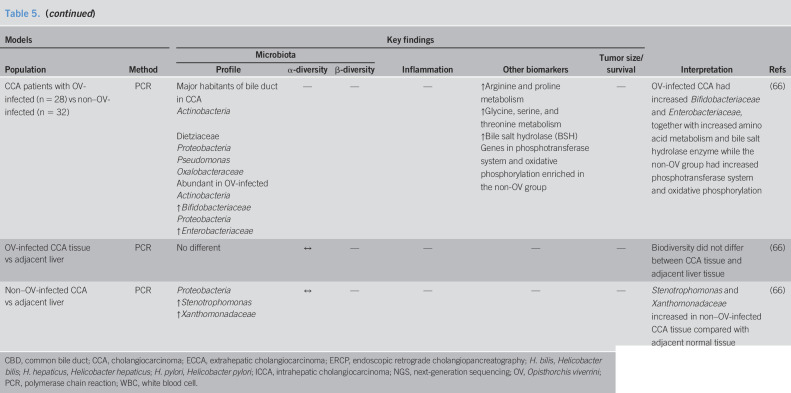
The roles of tissue microbiota changes in tumor model: reports from clinical studies

## CONCLUSION

Changes in microbiota in the gut, bile, and tumor site may play a significant role in CCA pathogenesis. A liver fluke OV infection could be the main factor contributing to dysbiosis. However, information regarding the association between microbiota profile and an OV infection in CCA patients is limited, and further investigation is needed. OV infection and dysbiosis in the gut, bile, and tumor have been shown to exacerbate chronic inflammation of cholangiocytes and bile acid metabolism changes, which could possibly lead to CCA development. Despite the variation in species abundance of microbiota among collected specimens, *Helicobactor* spp., *Bifidobacteriaceae*, and *Ruminococcaceae* were generally increased in CCA patients. Because of the limited reports available, in particular clinical studies, a number of questions related to the impact of changes in microbiota associated with CCA at various sites need further investigation. In addition, to better understand the association between gut, bile, and tumor microbiota in CCA pathogenesis, studies with rigorous experimental design and appropriate specimen collection in both basic and clinical settings are essential to establish therapeutic and prophylactic interventions in the future.

## CONFLICTS OF INTEREST

**Guarantor of the article:** Nipon Chattipakorn, MD, PhD.

**Specific author contributions:** T.K.: conceptual design, data collection, and drafting the manuscript. P.T., N.A., and S.C.C.: editing the manuscript. N.C.: conceptual design and editing the manuscript. All authors read and approved the final manuscript.

**Financial support:** This work was supported by the NSTDA Research Chair grant from the National Science and Technology Development Agency Thailand (N.C.), the Senior Research Scholar grant from the National Research Council of Thailand (S.C.C.), the Thailand Science Research and Innovation grant (N.C.), and the Chiang Mai University Center of Excellence Award (N.C.).

**Potential competing interests:** None to report.
